# Correction: Allosteric signalling in the outer membrane translocation domain of PapC usher

**DOI:** 10.7554/eLife.21585

**Published:** 2016-09-27

**Authors:** Irene Farabella, Thieng Pham, Nadine S Henderson, Sebastian Geibel, Gilles Phan, David G Thanassi, Anne H Delcour, Gabriel Waksman, Maya Topf

Farabella I, Pham T, Henderson NS, Geibel S, Phan G, Thanassi DG, Delcour AH, Waksman G, Topf M. 2014. Allosteric signalling in the outer membrane translocation domain of PapC usher. *eLife* **3**:e03532. doi: 10.7554/eLife.03532.

Published October 01, 2014

In the published article there was an error in the schematic representation of residues involved in the allosteric signalling to control PapC gating (Figure 7), showing the wrong shape (corresponding to the cellular phenotype) for two residues (C327 and K427).

The figure has been adjusted to show the correct shape, consistent with the results presented in the paper.

The corrected Figure 7 is shown here:

**Figure 1:**
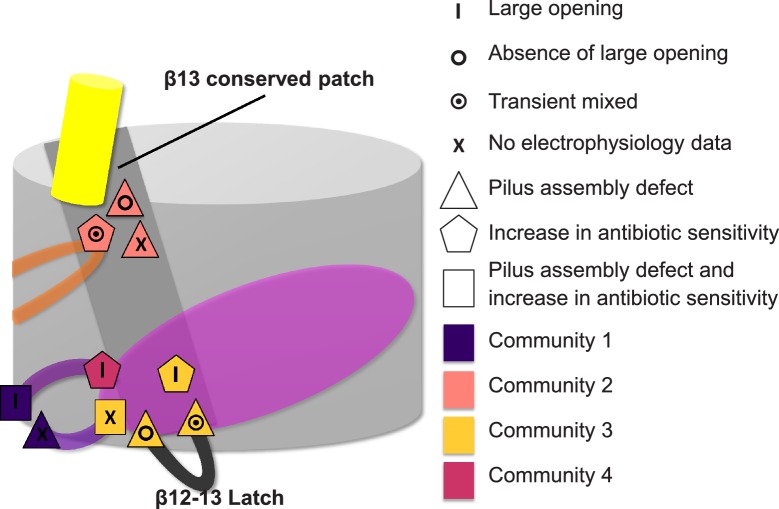

The originally published Figure 7 is also shown for reference:

**Figure 2:**
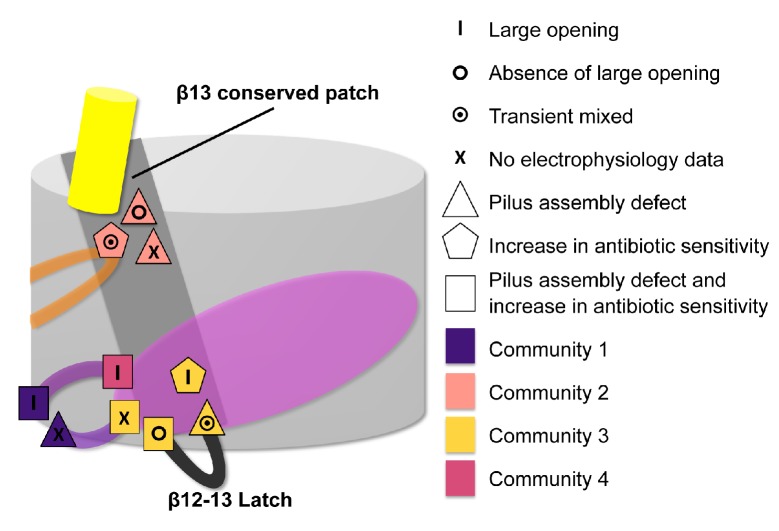

The article has been corrected accordingly.

